# Applying Monte Carlo Simulations to a Small Data Analysis of a Case of Economic Growth in COVID-19 Times

**DOI:** 10.1177/21582440231181540

**Published:** 2023-06-17

**Authors:** Nguyen Ngoc Thach

**Affiliations:** 1 Ho Chi Minh University of Banking, Vietnam

**Keywords:** Bayesian approach, frequentist estimation, small sample, economic growth, COVID-19

## Abstract

Studies on the going-on COVID-19 pandemic face small sample issues. In this context, Bayesian estimation is considered a viable alternative to frequentist estimation. Demonstrating the Bayesian approach’s advantage in dealing with this problem, our research conducted a case study concerning ASEAN economic growth during the COVID-19 pandemic. By using Monte Carlo standard errors and interval hypothesis testing to check parameter bias within a Bayesian MCMC simulation study, the author obtained significant conclusions as follows: first, in insufficient sample sizes, in contrast to frequentist estimation, the Bayesian framework can offer meaningful results, that is, expansionary monetary and contractionary fiscal policies are positively associated with economic growth; second, in the face of a small sample, by incorporating more information into prior distributions for the model parameters, Bayesian Monte Carlo simulations perform so far better than naïve Bayesian and frequentist estimation; third, in case of a correctly specified prior, the inferences are robust to different prior specifications. The author strongly recommends applying specific informative priors to Bayesian analyses, particularly in small sample investigations.

## Introduction

The COVID-19 outbreak, which took place in Wuhan (China) at the end of 2019, has been spreading worldwide at an extraordinary speed and scale. The COVID-19 shock severely damaged the ASEAN community (ADB, 2022). This unprecedented epidemic has become an enormous academic interest, with an increasing number of investigations conducted on its multidimensional impacts on the economies around the world. Recently, Padhan and Prabheesh (2021) provided a review of literature on the economics of the COVID-19 pandemic. Nevertheless, due to small sample sizes, most of the studies on the effects of the COVID-19 crisis derived conclusions relying mainly on conceptual arguments or descriptive data analyses (e.g., Alberola et al., 2021; Allen, 2021; Anand, 2020; Atkeson, 2020; Chetty et al., 2020; Curdia, 2020; Eichenbaum et al., 2020; Fornaro & Wolf, 2020), or frequentist econometric inferences with large data sets (e.g., Baqaee & Farhi, 2020; Chadha et al., 2020; Costa Junior et al., 2021; Faria E Castro, 2021; Iyke, 2020a; Jones et al., 2020; Jorda et al., 2020; Li et al., 2021). Data sample sizes needed for statistical inferences are insufficient to obtain unbiased, robust estimation results, especially in the case of one country or one small group of countries. The Bayesian approach is considered an alternative to more traditional frequentist estimation in small sample contexts.

Hence, the main goal of the current research is to conduct a case study in which the impacts of specific factors on GDP growth are estimated utilizing a small cross-section of 8 ASEAN nations to illustrate the advantages of Bayesian Markov chain Monte Carlo (MCMC) simulations over frequentist and even naïve Bayesian estimation. The results of the analyses suggest recommendations for using Bayesian methods in small sample investigations.

The remainder of the research is organized as follows: A review of literature on the COVID-19 effects and methodological and applied studies on the Bayesian paradigm is performed in Section 2. The methodology in the third section describes Bayesian MCMC simulation methods in which prior specifications are presented for a case study on ASEAN GDP growth. Section 4 demonstrates the Bayesian outcomes of the case study and interprets the results acquired. The fifth section concludes the research.

## Literature Review

The COVID-19 outbreak has raised academic interest in business cycle theory. A growing literature on the impacts of the COVID-19 disease has highlighted several aspects. The influence of the COVID-19 pandemic on financial markets was accessed by Al-Awadhi et al. (2020), Baker et al. (2020), Cao et al. (2021), Gil-Alana and Claudio-Quiroga (2020), Gormsen and Koijen (2020), Harjoto et al. (2021), D. Liu et al. (2020), Phan and Narayan (2020). Negative sentiments in connection with the COVID-19 pandemic have caused high volatility in the exchange rate of economies (Iyke, 2020b). Barro et al. (2020), Iyke (2020c), Choi (2020), Jorda et al. (2020), and L. Liu et al. (2020) concentrated attention on production and credit reduction during the COVID-19 period. There are plenty of studies on negative supply and demand shocks in the oil market (Apergis & Apergis, 2020; Devpura, 2020; Devpura & Narayan, 2020; Fu & Shen, 2020; Gil-Alana & Monge, 2020; Huang & Zheng, 2020; Kartal, 2021; Narayan, 2020; Polemis & Soursou, 2020; Qin et al., 2020). Also, there are a large number of analyses of monetary and fiscal policy instruments for fighting the COVID-19-driven recession (Alberola et al., 2021; Allen, 2021; Anand, 2020; Atkeson, 2020; Baqaee & Farhi, 2020; Chadha et al., 2020; Chetty et al., 2020; Costa Junior et al., 2021; Curdia, 2020; Eichenbaum et al., 2020; Faria E Castro, 2021; Fornaro & Wolf, 2020; Iyke, 2020b; Jones et al., 2020; Jorda et al., 2020; Li et al., 2021; Padhan & Prabheesh, 2021). The health impacts of the disease were analyzed by Najabat et al. (2021) The macroeconomic consequences of COVID-19 were assessed by McKibbin and Fernando (2020). Compared to the relatively rich body of literature on the COVID-19 impacts, studies on the influence of the COVID-19 pandemic on ASEAN economies are relatively scarce (e.g., Aziz et al., 2022; Gil-Alana & Monge, 2020; Hooi Hooi, 2022; JETRO, 2020; Kliem, 2021; Makun & Jayaraman, 2021; Morgan & Trinh, 2021; Purnomo et al., 2022; Rizvi et al., 2021; Sharma, 2020; Topcu & Gulal, 2020). Notably, most of the mentioned studies implemented descriptive analyses of data or frequentist modeling relying on sufficiently large data samples. In contrast, studies on COVID-19 impacts like ours face small sample issues due to a limited population.

Small data sets are often encountered in social science research for several reasons, such as the limitation of the research population (Roe-Sepowitz, 2009; van der Lee et al., 2008) or are hard to recruit and prone to drop-out (Mäkelä & Huhtanen, 2010; McCabe et al., 2016; Peeters et al., 2014), high costs of data acquisition (Price, 2012); ethical and moral constraints (van der Lee et al., 2008; van de Schoot et al., 2015), that all “make efforts to obtain a larger sample quite difficult (or impossible)” (Zondervan-Zwijnenburg et al., 2017). In small sample sizes, because of power issues, it is often impossible to acquire meaningful outcomes (Button et al., 2013; Lee & Song, 2004; Price, 2012). Many authors (Button et al., 2013; Kenward & Roger, 2009; Lee & Song, 2004; Natesan, 2015; Price, 2012; Smid et al., 2020; van de Schoot et al., 2015; Zondervan-Zwijnenburg et al., 2017, 2018) have stressed the power failure within the frequentist framework. Frequentist inference in small sample studies regularly leads to “non-convergence, inadmissible parameter solutions, and inaccurate estimates” (Smid et al., 2020), “low statistical power and level of relative bias” (B. O. Muthén & Curran, 1997; van de Schoot et al., 2015; Zondervan-Zwijnenburg et al., 2017). Non-significant p-values from underpowered estimations cannot offer a meaningful interpretation in the null hypothesis significance testing. As shown in comparative analyses, according to coverage levels for structural and variance parameters in small sample studies, least square (LS), maximum likelihood (ML), or restricted maximum likelihood (REML) perform worse than three Bayesian estimation methods with naïve, thoughtful (specific), and data-driven priors, respectively (Bradley, 1978); compared to ML, REML, and LS, the choice of thoughtful prior settings increases power for structural parameters in Bayesian estimation (Miočević et al., 2017; Price, 2012; van de Schoot et al., 2015; Zondervan-Zwijnenburg et al., 2018); Bayesian analysis with thoughtful and data-driven prior settings performs well for both parameter types, but bias level for variances is higher than structural parameters (Hoogland & Boomsma, 1998). Remarkably, the results from Bayesian inference using naïve prior settings are more biased than even data-driven frequentist inferences (Browne & Draper, 2000; Chen et al., 2014; Depaoli & Clifton, 2015; Holtmann et al., 2016; D. M. McNeish, 2016). One main reason behind a high bias level in naïve Bayesian estimation is the priors’ relatively more significant effect on posteriors in the case of small sample sizes and complex models (Lee & Song, 2004; D. M. McNeish, 2016; Natesan, 2015). Diffuse or vague prior distributions give a vast range of plausible parameter values that can be sampled in the MCMC iterations. The probability mass, as a consequence, often lies on outliers. More importantly, Bayesian inference with thoughtful prior settings performs better than frequentist and naïve Bayesian inference in most simulation studies (Depaoli & Clifton, 2015; D. M. McNeish, 2016; Miočević et al., 2017; Natesan, 2015; Price, 2012; Serang et al., 2015; van de Schoot et al., 2015; Yuan & MacKinnon, 2009; Zondervan-Zwijnenburg et al., 2018) whereas only some authors gave a preference for one of the two approaches depending on the quantity or precision of information captured in priors (Depaoli, 2012, 2013; Holtmann et al., 2016; D. M. McNeish, 2016), or prior choice (Baldwin & Fellingham, 2013).

Frequentists often ask, “how large must a data set be?” and can find a solution to this problem in ratios of the number of parameters to the sample size in a research model. Lee and Song (2004) suggested that for structural equation models, to generate reliable results from ML estimation, this ratio should be 1:5, while a ratio of 1:3 might yield some parameter bias. The Bayesian analysis does not require large data sets, as with frequentist methods. A Bayesian simulation study assumes a much smaller ratio of parameters to observations. A 1:3 ratio can be used instead of 1:5. The use of Bayesian modeling allows us to circumvent all the problems mentioned above as recommended by Bayesians (e.g., B. Muthén & Asparouhov, 2012; Smid et al., 2020; Wagenmakers et al., 2008). In Bayesian inference, a posterior distribution resulting from a combination of a prior distribution with the data distribution is demonstrated as a distribution representative of the probability of parameter values.

Throughout the current research, a cross-section concerning ASEAN economic growth is analyzed as a case study to display the advantages the Bayesian approach has to handling inadequate sample issues. In the case study, with the inclusion of featured variables in a multivariate regression model based on a dataset of 8 ASEAN nations (excluding Laos, Cambodia, and Timor-Leste for lack of data), the adoption of frequentist estimators can lead to biased, variable, and unreliable outcomes. Hence, a Bayesian approach via Monte Carlo simulations needs to be employed instead to obtain meaningful outcomes. The study is expected to achieve the following conclusions: first, in small sample sizes, compared to frequentist and naïve Bayesian estimation, a thoughtful Bayesian approach can generate meaningful results; second, in small sample studies, the more information is available in the priors for the parameters in the model, the more precise and robust the posterior results become.

## Methodology

### Monte Carlo Simulations in Bayesian Perspective

Since the 1990s, we have witnessed a rapid increase in the application of Bayesian statistical methods in a variety of scientific areas (e.g., Hung et al., 2019; König & van de Schoot, 2018; Rietbergen et al., 2017; Thach et al., 2021, 2022, 2022). A sharp rise in both methodological and applied elaboration of the Bayesian approach has been observed in economics. That is thanks to recent advancements in software programs and significant advantages the Bayesian approach has over the frequentist framework, such as the capability of solving too complex models or models too demanding for frequentist methods; straightforward probability interpretations of estimation results; application of simple probability rule, Bayes’ law to all models; the flexibility to involve model uncertainty (Kaplan, 2014; van de Schoot et al., 2017; Wagenmakers et al., 2008). One of the superior features of Bayesian inference over frequentist inference is that the former is not based on asymptotic theory. A combination of prior information with available data results in a posterior distribution that presents a probabilistic distribution of parameter values irrespective of sample size. Prior distributions contain knowledge about model parameters before observing the data. If no information is available, default or non-informative priors are often chosen that specify a wide range of parameter values. In such a way, however, the Bayesian approach will get rid of its main advantages. As a rule, researchers tend to limit the admissible parameter space by specifying informative priors. With such a prior specification, the accuracy of the posterior distribution can increase. When more information is incorporated into a prior distribution, it becomes narrower, so the posterior distribution is influenced more by the prior information. In that case, an increase in statistical power might cause less parameter bias. To summarize, Bayesian inference with informative priors, no matter the sample size, can produce meaningful results, and the precision of the conclusion increases if more information is included. In particular, a thoughtful Bayesian approach allows for mitigating statistical issues related to frequentist inference, such as multicollinearity (Block et al., 2011).

Smid et al. (2020) categorized priors into three types for Bayesian inference: naïve, thoughtful (specific), and data-driven, and there are various levels of informativeness and various combinations of naïve, thoughtful, and data-driven priors within each of these Bayesian categories. The prior setting is viewed as thoughtful in case at least one prior distribution contains information, often used in conjunction with default priors (Smid et al., 2020). Naïve priors rest on software defaults, or generalized suggestions, while data-driven priors are derived from the output of LS, ML, REML, or naïve Bayesian estimation. Smid et al. (2020) emphasized that studies applying naïve or data-driven priors are justifiable under some circumstances; they can be used as thoughtful priors.

The superiority of thoughtful priors over naïve priors is documented in most of the simulation studies (Chen et al., 2014; Depaoli & Clifton, 2015; Holtmann et al., 2016; D. M. McNeish, 2016; Miočević et al., 2017; Natesan, 2015; Price, 2012; Serang et al., 2015; van de Schoot et al., 2015; Yuan & MacKinnon, 2009; Zondervan-Zwijnenburg et al., 2018). Posterior distributions become less biased and more accurate than frequentist and naïve Bayesian methods when more prior information is added, mainly when the hyperparameters of a prior distribution are similar to the population values. In the thoughtful Bayesian category, the level of informativeness is varied by adjusting the variance hyperparameter of the prior distribution (Depaoli, 2012, 2013; Depaoli & Clifton, 2015; Holtmann et al., 2016; van de Schoot et al., 2015; Zondervan-Zwijnenburg et al., 2018) or by adjusting both the hyperparameters (D. M. McNeish, 2016; Natesan, 2015). Based on weakly informative priors, Bayesian performance might still be poor for large variance hyperparameters are specified. But, adding strongly informative priors improves the results in terms of power and relative bias (Depaoli, 2012; Holtmann et al., 2016; D. M. McNeish, 2016).

In the case of lacking knowledge about model parameters, Darnieder (2011) recommended selecting data-driven priors for small sample analyses. Lee and Song (2004), D. M. McNeish (2016), and van Erp et al. (2018) reported that Bayesian results based on data-driven priors are better than those from frequentist and naïve Bayesian estimations.

Variance parameters often have more bias than structural parameters (D. M. McNeish, 2016). B. Muthén and Asparouhov (2012) stated that the prior for variance terms is substantial for small sample sizes. Furthermore, in Bayesian modeling, with the use of half-Cauchy or Inverse Gamma priors, variance parameters give better results than other priors (Gelman, 2006; D. McNeish & Stapleton, 2016). van de Schoot et al. (2015) advised using non-informative Inverse Gamma (0.001, 0.001) or very informative Inverse Gamma (0.5, 0.5) for variance components.

**Figure 1.**
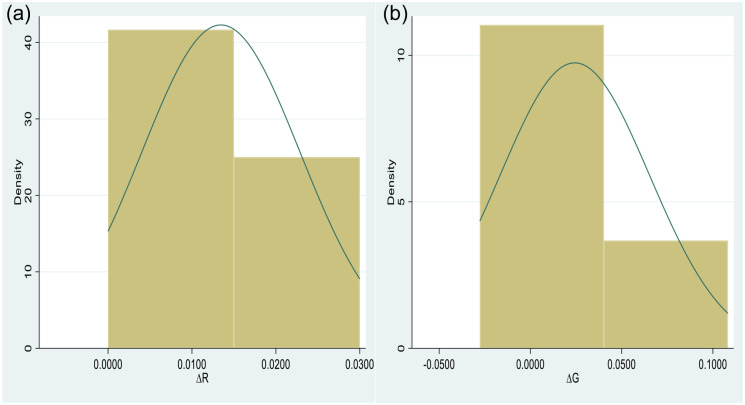
The density of the parameters of interest. (a) For ΔR and (b) For ΔG.

All the analyses presented above assist us in designing a simulation strategy for this research. First, to identify our model’s distributions of structural parameters, we plot the density of all the variables. They are all normally distributed. Figure 1a and b display the normal distribution of the parameters of interest, ΔR and ΔG. These regression parameters are normally distributed with mean and variance hyperparameters. In the second step, emphasizing policy responses to the economic growth of the ASEAN countries, we assign informative priors to the two parameters. At the same time, default prior settings are specified for the remaining variables. The mean values are extracted from LS estimates as well-specified priors in our context (van de Schoot et al., 2015). Although the Bayesian framework is preferable to tackle the inadequate sample issues, simply switching to the Bayesian approach can generate a worse result than the frequentist framework when a small sample model is fit without due diligence to ensure that outcomes are as trustworthy as possible. So, a sensitivity analysis needs to be performed to check how varying values for prior variance influence the posterior distribution. For our case study, the prior variance can be varied in descending order (10,000,1,000, 100, 50, 20, 10, 5, 3, 1). A variance of10,000 is the default prior setting. A variance of 100 is still considered non-informative, whereas a variance of 1 is strongly informative. However, different from the previous studies (Smid et al., 2020; van de Schoot et al., 2015), along with standard deviations, Monte Carlo standard errors are used as a measure of bias, and interval hypothesis testing serves to determine parameter significance in this research. The third step suggests varying both the prior mean and the prior variance. For illustrative purposes, a misspecified prior mean of −5, vastly distinguished from the well-specified one, can be chosen while the prior variance is varied as in the above. Another sensitivity analysis is carried out to check how the misspecified prior distribution influences the posterior distribution when the prior variance ranges from10,000 to 1. According to Bayesians (Gelman, 2006; D. M. McNeish, 2016; D. McNeish & Stapleton, 2016; van de Schoot et al., 2015), variance terms are often problematic, so we set Inverse Gamma distributions of (0.01, 0.01) (non-informative) and (0.5, 0.5) (strongly informative) for the overall variance and perform a sensitivity analysis through a convergence test. Last, we choose the most suitable model to run MCMC simulations and draw inferences.

### Model and Data

For this simulation design, we fit a Bayesian regression model:

$$\begin{array}{c} \mathrm{Grow}=\beta_{1}+\beta_{2}\mathrm{Gro}w_{-1}+\beta_{3}\mathrm{OT}+\beta_{4}\mathrm{Int}+\beta_{5}\mathrm{Cov}\\ \;+\beta_{6}\Delta R+\beta_{7}\Delta G+u. \end{array}$$

In the case study, the data utilized to estimate the specific effects on GDP growth in times of the COVID-19 pandemic are a cross-country sample of 8 ASEAN nations (Brunei, Indonesia, Myanmar, Malaysia, the Philippines, Singapore, Thailand, and Vietnam). The point of time is 2021, except for variable Int with its updated data available only to the year 2020. Notice that because observations and parameters reach the same amount of 8, a 1:1 ratio is accessed; as defined by Lee and Song (2004), we obtain a too-small data set. The specific factors of GDP growth that are selected for the case study are variables lagged GDP, trade openness, internet penetration rate, coronavirus infection rate, and monetary and fiscal policy instruments as used in Thach, Linh et al. (2022). Still, the key difference is that this research selects decreases in the interest rate and government expenditure as proxies for policy decisions (Table 1).

**Table 1.** Explaining Model Variables.

Variables		Proxies	Notation	Data source
Dependent	Economic growth in the year 2021	Real GDP growth in the year 2021 (%)	Grow	World Bank (2022)
Independent	Economic growth in the year 2020	Real GDP growth in the year 2020 (%)	$\mathrm{Gro}w_{-1}$	World Bank (2022)
	Trade openness	Percentage of trade to GDP (%)	OT	World Bank (2022)
	Internet penetration rate	Percentage of number of internet users to population (%)	Int	World Bank (2022)
	Coronavirus infection rate	Percentage of number of COVID-19 infections to population (%)	Cov	Worldometer (2021)
	Expansionary monetary policy	Decrease in the central bank policy rate	ΔR	IMF (2022a)
	Contractionary fiscal policy	Decrease in government expenditure (% of GDP)	ΔG	IMF (2022b)

The simulation study uses a target MCMC sample size of 10,000, with the first 2,500 burn-in iterations discarded from the MCMC sample. To check chain convergence, the author set a thinning of 10 and simultaneously increased the MCMC sample size to 50,000. The total amount of iterations for the Metropolis-Hastings sampling is 102,491. Note that checks for sequence convergence have to be conducted using a Monte Carlo technique before the inferential stage. Once the MCMC chain has converged to a stationary distribution, resulting inferences become reliable.

## Bayesian Simulation Outcomes

**Figure 2.**
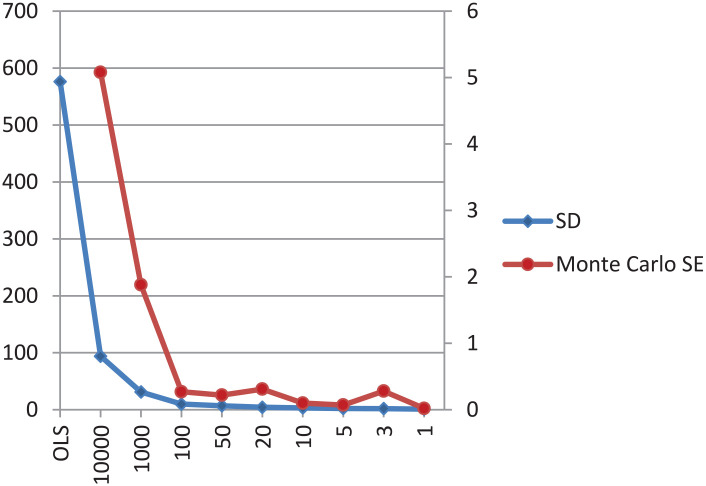
Influence of OLS versus Bayesian methods and various values for prior variance, but with a fixed mean for ΔR. *Source*. The author’s calculation. *Note*. The left and right y-axes express frequentist SD and Monte Carlo SE, respectively; the x-axis displays the various values for the prior variance for ΔR (and ΔG).

**Figure 3.**
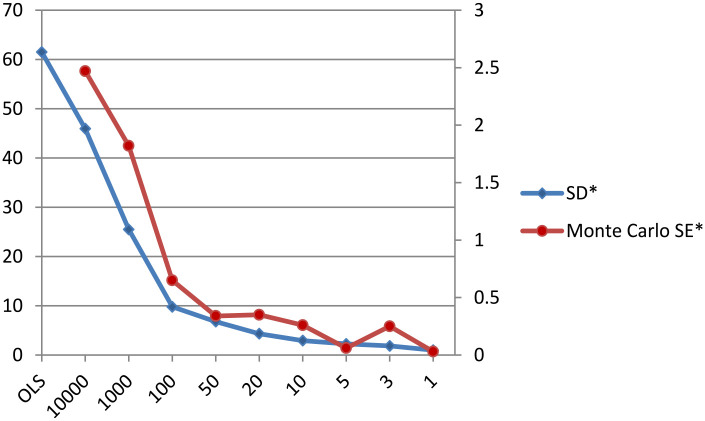
Influence of OLS versus Bayesian methods and various values for prior variance, but with a fixed mean for ΔG. *Source*. the author’s calculation. *Note*. The x-axis represents the various values for the prior variance for ΔG (and ΔR).

By default, the structural parameters are normally distributed with hyperparameters of (0,10,000), a non-informative prior setting. An inverse gamma distribution with hyperparameters of (0.01, 0.01) was specified by default for the variance parameter. Concerning the parameters of interest, ΔR and ΔG, to examine the influence of the prior specification on the posterior distribution, we implemented the first sensitivity analysis with the decreasing values for the prior variance (10000–1). Default prior settings were specified for the rest of the parameters in the model. The effects of different specifications of the prior variance on the posterior standard deviation (SD) and the Monte Carlo standard error (SE) for ΔR and ΔG with the fixed prior means of 492 and 35, respectively, are demonstrated in Figures 2 and 3. It is clearly shown that the narrower the specified prior variance, the lower both posterior SD and Monte Carlo SE.

It is concluded that in case a well-specified prior mean is included in a small sample simulation study, the more information is captured in the prior variance, the more accurate the posterior estimates become (i.e., estimates of SD and Monte Carlo SE become lower), and, more importantly, thoughtful Bayesian estimation outperforms both naïve Bayesian and frequentist estimation in terms of bias. Besides, the author makes one more immediate conclusion that with the well-specified prior setting, the results remain robust across the different specifications for the prior variance; that is, the various values for the prior variance do not change the posterior estimates.

**Figure 4.**
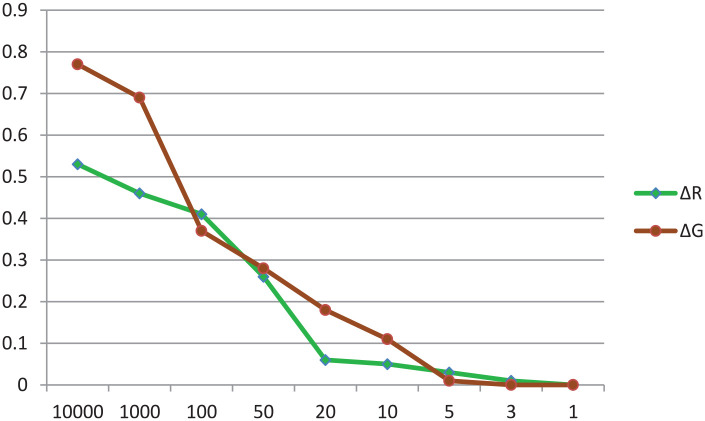
Probability of posterior estimates being significant in the case of a misspecified prior mean (=−5) for ΔR and ΔG. *Source*. The author’s calculation.

To analyze the effects of potential misspecification of the prior mean, both the hyperparameters of the normal distribution for ΔR and ΔG were varied. Instead of the well-specified prior mean, a prior mean of −5 and, at the same time, a wide range of values for the prior variance (1000–1) were set on the parameters ΔR and ΔG. The results of the second sensitivity analysis are exhibited in Figure 4. The various values for the prior variance are displayed on the x-axis and probability of the posterior distribution on the y-axis. As is visible, the narrower the value for the prior variance, the lower the probability of the posterior estimates. From the sensitivity analysis results, we can conclude that in the case of misspecification of the prior mean, the more information captured in the prior variance, the more biased the posterior estimates become. In some cases, there may even be changes in the results.

**Figure 5.**
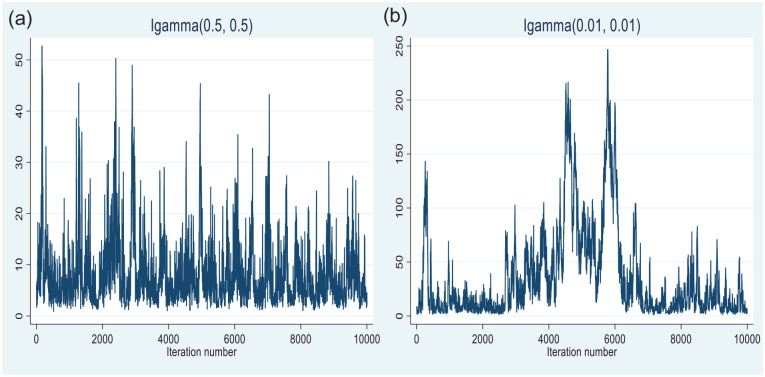
Trace plot for the overall variance of the gamma distribution, IG(0.5, 0.5) (a) and inverse gamma distribution, IG(0.01, 0.01) (b). *Source*. The author’s calculation.

The biased results for the overall variance can be clarified by testing trace plots. As is demonstrated in Figure 5a and b, trace plots for the overall variance parameter with an inverse gamma distribution, IG (0.5, 0.5), look better than those with an inverse gamma distribution, IG(0.01, 0.01). IG(0.5, 0.5) is a too-informative prior distribution, while IG(0.01, 0.01) is the default setting. According to the trace plots, since the Markov chain has converged for IG(0.5, 0.5), we chose this probability distribution for the prior variance of the model. As a result, the appropriate empirical model for our analysis has the normal distributions with the hyperparameters N(492, 1) and N(35, 1) for ΔR and ΔG, respectively, and an inverse gamma distribution, IG(0.5, 0.5) for the overall variance parameter. *An immediate conclusion is that the prior specifications of the overall variance influence the estimation of the posterior variance.*

**Figure 6.**
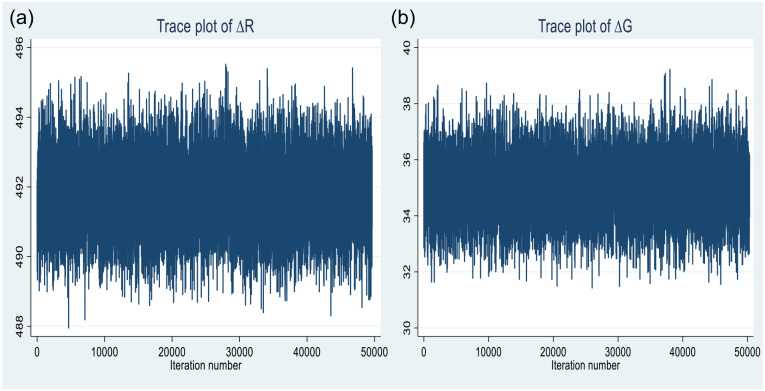
Trace plots of ΔR (a) and ΔG (b).

According to the estimation results, an acceptance rate of 6% and average efficiency of 4% are admissible for a Monte Carlo simulation algorithm (Roberts & Rosenthal, 2001). The trace plots for all the model parameters are verified for anomalies, and none are revealed. As the trace plots for ΔR and ΔG are exhibited in Figure 6a and b.

In sum, a conclusion derived from all the analyses above is that the Bayesian Monte Carlo simulation method relying on the informative prior settings could yield meaningful posterior estimates in small samples. Our results are consistent with Yuan and MacKinnon (2009), Price (2012), Depaoli and Clifton (2015), Natesan (2015), Chen et al. (2014), van de Schoot et al. (2015), Serang et al. (2015), Holtmann et al. (2016), D. M. McNeish (2016), Miočević et al. (2017), and Zondervan-Zwijnenburg et al. (2018).

The estimation summary recorded in Table 2 indicates that the posterior estimates are acceptably accurate and robust. Regarding probability, PPIs for ΔR and ΔG do not contain zero, implying that changes in the central bank interest rate and government expenditure strongly impact GDP growth (proxied by Grow). A reasonable explanation for the contractionary fiscal policy is that most government expenditure is non-productive and used almost inefficiently during COVID-19. Similarly, the internet penetration rate (proxied by Int) is strongly positively correlated to GDP growth, while the COVID-19 infection rate (proxied by Cov) is strongly negatively correlated to GDP growth. However, on the contrary, the effects of trade openness (proxied by OT) and the lagged GDP (proxied by Grow-1) are ambiguous.

**Table 2.** Posterior Estimates With Specific Priors.

Variables	Reg. coef.	Posterior SD	Monte Carlo SE	PPI
Grow				
$\mathrm{Gro}w_{-1}$	−0.237	0.182	0.005	−0.598 to 0.139
OT	−0.805	1.675	0.051	−4.076 to 2.677
Int	33.417	10.282	0.324	11.421 to 53.319
ΔR	492.05	1.012	0.027	490.043 to 493.993
Cov	−18.754	6.688	0.140	−32.096 to −4.844
ΔG	35.010	1.001	0.013	33.062 to 36.991
Intercept	−26.291	5.283	0.173	−36.345 to −14.976
Overall variance	10.703	13.803	0.389	1.995 to 46.496

*Source*. The author’s calculation.

*Note*. SD = standard deviation; SE = standard error; PPI (Posterior probability interval) = the 95% probability that a parameter lies between two values in the population.

## Concluding Remarks

By implementing a case study on ASEAN economic growth, the current research showed that in a small sample investigation, with the assignment of specific prior settings, Bayesian performance is better than both naïve Bayesian and frequentist performance, and more importantly, the Bayesian results are meaningful. Furthermore, the more information incorporated in the prior distributions, the less biased and the more precise the posterior estimates become. By using Monte Carlo standard errors to measure the level of bias and interval hypothesis testing to check parameter significance, the outcomes show that a model with highly informative priors is the best fit for statistical inferences in a small sample context. Based on the outcomes, the author derives some generalized conclusions as follows. In modeling small sample data, thoughtful (specific) Bayesian estimation outperforms naïve Bayesian and frequentist estimation. Besides, in small sample sizes, Bayesian inferences are highly sensitive to prior specifications of the model parameters: with specifying default prior or diffuse settings, naïve Bayesian estimation can produce even more biased inferences than frequentist estimation; but, with the correctly specified prior hyperparameters, the posterior distributions become more accurate (less biased) and more robust (less variable) when more information is added to the prior distributions. From the outcomes acquired in this study, the author strongly recommends users of Bayesian methods adopt thoughtful priors in research, regardless of data sample size. Additionally, some policy implications are provided: first, an expansionary monetary policy is needed in a crisis, as Keynesians suggest. However, fiscal policy needs to focus on productive government spending, while non-productive expenses should be adequate; second, in the current information technology era, the Internet is also one of the essential factors for economic, cultural, and social development and so increases in internet penetration are necessary; third, infection and death cases caused by the COVID-19, reducing labor force, adversely affect economic growth, so healthcare measures should be implemented efficiently.

Limitation and future research direction: This study used data-driven priors for the variables of interest. Although Bayesian estimation with priors derived from performs better than frequentist and naïve Bayesian estimation, a future Bayesian simulation study will have a more exciting result if the researchers specify informative priors rather than data-driven priors.

## References

[bibr1-21582440231181540] ADB. (2022). Southeast Asia rising from the pandemic. 10.22617/TCS220093-2.

[bibr2-21582440231181540] Al-Awadhi A. M. Alsaifi K. Al-Awadhi A. Alhammadi S. (2020). Death and contagious infectious diseases: Impact of the COVID-19 virus on stock market returns. Journal of Behavioral and Experimental Finance, 27, 100326. 10.1016/j.jbef.2020.100326 32292707 PMC7144859

[bibr3-21582440231181540] Alberola E. Arslan Y. Cheng G. Moessner R. (2021). Fiscal response to the COVID-19 crisis in advanced and emerging market economies ^†^. Pacific Economic Review, 26(4), 459–468. 10.1111/1468-0106.12370

[bibr4-21582440231181540] Allen W. A. (2021). Monetary Policy and government debt management during the Coronavirus Pandemic. National Institute Economic Review, 255, 79–84.

[bibr5-21582440231181540] Anand P. (2020). Economic Policies for COVID-19, IZA Policy Papers, 156, Institute of Labor Economics (IZA). https://docs.iza.org/pp156.pdf.

[bibr6-21582440231181540] Apergis E. Apergis N. (2020). Can the COVID-19 pandemic and oil prices drive the US partisan conflict index? Energy Research Letters, 1(1), 13144. 10.46557/001c.13144

[bibr7-21582440231181540] Atkeson A. (2020). What Will Be the Economic Impact of COVID-19 in the US? Rough Estimates of Disease Scenarios (NBER Working Papers no 26867). National Bureau of Economic Research, Inc. https://www.nber.org/system/files/working_papers/w26867/w26867.pdf

[bibr8-21582440231181540] Aziz M. I. A. Ahmad N. Zichu J. Nor S. M. (2022). The impact of COVID-19 on the connectedness of stock index in ASEAN+3 economies. Mathematics, 10, 1417. 10.3390/math10091417

[bibr9-21582440231181540] Baker S. R. Bloom N. Davis S. J. Kost K. J. Sammon M. C. Viratyosin T. (2020). The unprecedented stock market impact of COVID-19 (NBER Working Paper no 26945). https://www.nber.org/papers/w26945.

[bibr10-21582440231181540] Baldwin S. A. Fellingham G. W. (2013). Bayesian methods for the analysis of small sample multilevel data with a complex variance structure. Psychological Methods, 18(2), 151–164. 10.1037/a0030642 23148476

[bibr11-21582440231181540] Baqaee D. Farhi E. (2020). Supply and Demand in Disaggregated Keynesian Economies with an Application to the Covid-19 Crisis (NBER Working Paper Series no 27152), National Bureau of Economic Research, Cambridge, MA.

[bibr12-21582440231181540] Barro R. J. Ursúa J. F. Weng J. (2020). The coronavirus and the great influenza pandemic: Lessons from the spanish flu for the coronavirus’s potential effects on mortality and economic activity (NBER Working Paper no 26866). https://www.nber.org/papers/w26866.

[bibr13-21582440231181540] Block J. H. Jaskiewicz P. Miller D. (2011). Ownership versus management effects on performance in family and founder companies: A Bayesian reconciliation. Journal of Family Business Strategy, 2, 232–245.

[bibr14-21582440231181540] Bradley J. V. (1978). Robustness? British Journal of Mathematical and Statistical Psychology, 31(2), 144–152. 10.1111/j.2044-8317.1978.tb00581.x 737141

[bibr15-21582440231181540] Browne W. J. Draper D. (2000). Implementation and performance issues in the Bayesian and likelihood fitting of multilevel models. Computational Statistics, 15(3), 391–420. 10.1007/s001800000041

[bibr16-21582440231181540] Button K. S. Ioannidis J. P. Mokrysz C. Nosek B. A. Flint J. Robinson E. S. Munafò M. R. (2013). Power failure: Why small sample size undermines the reliability of neuroscience. Nature Reviews Neuroscience, 14(5), 365–376. 23571845 10.1038/nrn3475

[bibr17-21582440231181540] Cao K. H. Li Q. Liu Y. Woo C. K. (2021). Covid-19’s adverse effects on a stock market index. Applied Economics Letters, 28, 1157–1161. 10.1080/13504851.2020.1803481

[bibr18-21582440231181540] Chadha J. S. Corrado L. Meaning J. Schuler T. (2020). Bank reserves and broad money in the global _nancial crisis: a quantitative evaluation (ECB Working Paper Series no 2463).

[bibr19-21582440231181540] Chen J. Choi J. Weiss B. A. Stapleton L. (2014). An empirical evaluation of mediation effect analysis with manifest and latent variables using Markov chain Monte Carlo and alternative estimation methods. Structural Equation Modeling, 21(2), 253–262. 10.1080/10705511.2014.882688

[bibr20-21582440231181540] Chetty R. Friedman J. Hendren N. (2020). How Did COVID-19 and Stabilization Policies Affect Spending and Employment? A New Real-Time Economic Tracker Based on Private Sector Data (NBER Working Papers no 27431). 10.3386/w27431

[bibr21-21582440231181540] Choi S. Y. (2020). Industry volatility and economic uncertainty due to the COVID-19 pandemic: Evidence from wavelet coherence analysis. Finance Research Letters, 37, 101783. 10.1016/j.frl.2020.101783 33013239 PMC7524523

[bibr22-21582440231181540] Costa Junior C. J. Garcia-Cintado A. C. Junior K. M. (2021). Macroeconomic policies and the pandemic-driven recession. International Review of Economics & Finance, 72(C), 438–465. 10.1016/j.iref.2020.12.010

[bibr23-21582440231181540] Curdia V. (2020). Mitigating COVID-19 effects with conventional monetary policy. FRBSF Economic Letter, 9, https://www.frbsf.org/economic-research/files/el2020-09.pdf

[bibr24-21582440231181540] Darnieder W. F. (2011). Bayesian methods for data-dependent priors [Doctoral dissertation, The Ohio State University]

[bibr25-21582440231181540] Depaoli S. (2012). Measurement and structural model class separation in mixture CFA: ML/EM versus MCMC. Structural Equation Modeling, 19(2), 178–203. 10.1080/10705511.2012.659614

[bibr26-21582440231181540] Depaoli S. (2013). Mixture class recovery in GMM under varying degrees of class separation: Frequentist versus Bayesian estimation. Psychological Methods, 18(2), 186–219. 10.1037/a0031609 23527607

[bibr27-21582440231181540] Depaoli S. Clifton J. P. (2015). A Bayesian approach to multilevel structural equation modeling with continuous and dichotomous outcomes. Structural Equation Modeling, 22(3), 327–351. 10.1080/10705511.2014.937849

[bibr28-21582440231181540] Devpura N. (2020). Can oil prices predict Japanese yen? Asian Economics Letters, 1(3), 17964. 10.46557/001c.17964

[bibr29-21582440231181540] Devpura N. Narayan P. K. (2020). Hourly oil price volatility: The role of COVID-19. Energy Research Letters, 1(2), 13683. 10.46557/001c.13683

[bibr30-21582440231181540] Eichenbaum M. S. Rebelo S. Trabandt M. (2020). The macroeconomics of epidemics (NBER Working Paper no 26882). https://www.nber.org/system/files/working_papers/w26882/w26882.pdf.

[bibr31-21582440231181540] Faria E Castro M. (2021). Fiscal Policy during a pandemic (Federal Reserve Bank of St. Louis Working Paper, 2020–3006). 10.20955/wp.2020.006

[bibr32-21582440231181540] Fornaro L. Wolf M. (2020). Covid-19 Coronavirus and Macroeconomic Policy (Working Papers no 1168), Barcelona Graduate School of Economics. https://bse.eu/research/working-papers/covid-19-coronavirus-and-macroeconomic-policy.

[bibr33-21582440231181540] Fu M. Shen H. (2020). COVID-19 and corporate performance in the energy industry. Energy Research Letters, 1(1), 12967. 10.46557/001c.12967

[bibr34-21582440231181540] Gelman A. (2006). Prior distributions for variance parameters in hierarchical models (Comment on article by browne and draper). Bayesian Analysis, 1(3), 515–534. 10.1214/06-BA117A

[bibr35-21582440231181540] Gil-Alana L. A. Claudio-Quiroga G. (2020). The COVID-19 impact on the Asian stock markets. Asian Economics Letters, 1(2), 17656. 10.46557/001c.17656

[bibr36-21582440231181540] Gil-Alana L. A. Monge M. (2020). Crude oil prices and COVID-19: Persistence of the shock. Energy Research Letters, 1(1), 13200. 10.46557/001c.13200

[bibr37-21582440231181540] Gormsen N. J. Koijen R. S. (2020). Coronavirus: Impact on stock prices and growth expectations (Working Paper no 22), University of Chicago, Becker Friedman Institute for Economics. 10.2139/ssrn.3555917.

[bibr38-21582440231181540] Harjoto M. A. Rossi F. Paglia J. K. (2021). COVID-19: Stock market reactions to the shock and the stimulus. Applied Economics Letters, 28, 795–801. 10.1080/13504851.2020.1781767

[bibr39-21582440231181540] Holtmann J. Koch T. Lochner K. Eid M. (2016). A comparison of ML, WLSMV, and Bayesian methods for multilevel structural equation models in small samples: A simulation study. Multivariate Behavioral Research, 51(5), 661–680. 10.1080/00273171.2016.1208074 27594086

[bibr40-21582440231181540] Hoogland J. J. Boomsma A. (1998). Robustness studies in covariance structure modeling. Sociological Methods & Research, 26(3), 329–367.

[bibr41-21582440231181540] Hooi Hooi L . (eds) (2022). Revitalising ASEAN Economies in a Post-COVID-19, World: Socioeconomic Issues in the New Normal. 10.1142/12046.

[bibr42-21582440231181540] Huang W. Zheng Y. (2020). COVID-19: Structural changes in the relationship between investor sentiment and crude oil futures price. Energy Research Letters, 1(2), 13685. 10.46557/001c.13685

[bibr43-21582440231181540] Hung T. Nguyen Trung N.D Thach N.N. (2019). Beyond traditional probabilistic methods in econometrics. In Kreinovich V. Thach N. Trung N. Van Thanh D. (Eds.), Beyond traditional probabilistic methods in economics. ECONVN 2019. Studies in Computational Intelligence (Vol. 809, pp. 3–21). Springer.

[bibr44-21582440231181540] IMF. (2022a). WEO 2022. https://www.imf.org/en/Publications/WEO/weo-database/2022/April.

[bibr45-21582440231181540] IMF. (2022b). International Financial Statistics.

[bibr46-21582440231181540] Iyke B. N. (2020a). COVID-19: The reaction of US oil and gas producers to the pandemic. Energy Research Letters, 1(2), 13912. 10.46557/001c.13912

[bibr47-21582440231181540] Iyke B. N. (2020b). Economic policy uncertainty in times of COVID-19 pandemic. Asian Economics Letters, 1(2), 17665. 10.46557/001c.17665

[bibr48-21582440231181540] Iyke B. N. (2020c). The disease outbreak channel of exchange rate return predictability: Evidence from COVID-19. Emerging Markets Finance and Trade, 56(10), 2277–2297. 10.1080/1540496X.2020.1784718

[bibr49-21582440231181540] JETRO. (2020). Impact of COVID-19 on Supply Chains in the ASEAN Plus Three Region, with Policy Recommendations ASEAN Plus Three Joint Study - Sub-report of Japan. https://www.jetro.go.jp/ext_images/en/reports/survey/pdf/COVID-19_202011.pdf.

[bibr50-21582440231181540] Jones C. J. Philippon T. Venkateswaran V. (2020). Optimal mitigation policies in a pandemic: Social distancing and working from home (NBER working papers no 26984), National Bureau of Economic Research, Inc. https://www.nber.org/system/files/working_papers/w26984/w26984.pdf

[bibr51-21582440231181540] Jorda O. Singh S. R. Taylor A. M. (2020). Longer-run economic consequences of pandemics (NBER Working Paper no 26934). https://www.nber.org/papers/w26934.

[bibr52-21582440231181540] Kaplan D. (2014). Bayesian statistics for the social sciences. The Guilford Press.

[bibr53-21582440231181540] Kartal M. T. (2021). The effect of the COVID-19 pandemic on oil prices: Evidence from Turkey. Energy Research Letters, 1(4), 18723. 10.46557/001c.18723

[bibr54-21582440231181540] Kenward M. G. Roger J. H. (2009). An improved approximation to the precision of fixed effects from restricted maximum likelihood. Computational Statistics & Data Analysis, 53(7), 2583–2595. 10.1016/j.csda.2008.12.013

[bibr55-21582440231181540] Kliem F. (2021). ASEAN and the EU amidst COVID-19: Overcoming the self-fulfilling prophecy of realism. Asia Europe Journal, 19(3), 371–389. 10.1007/s10308-021-00604-8 33746661 PMC7954680

[bibr56-21582440231181540] König C. van de Schoot R. (2018). Bayesian statistics in educational research: A look at the current state of affairs. Educational Review, 70, 486–509. 10.1080/00131911.2017.1350636

[bibr57-21582440231181540] Lee S. Y. Song X. Y. (2004). Evaluation of the Bayesian and maximum likelihood approaches in analyzing structural equation models with small sample sizes. Multivariate behavioral research, 39(4), 653–686. 26745462 10.1207/s15327906mbr3904_4

[bibr58-21582440231181540] Liu D. Sun W. Zhang X. (2020). Is the Chinese economy well positioned to fight the COVID-19 pandemic? The financial cycle perspective. Emerging Markets Finance and Trade, 56(10), 2259–2276. 10.1080/1540496X.2020.1787152

[bibr59-21582440231181540] Liu L. Wang E. Z. Lee C. C. (2020). Impact of the COVID-19 pandemic on the crude oil and stock markets in the US: A time-varying analysis. Energy Research Letters, 1(1), 13154. 10.46557/001c.13154

[bibr60-21582440231181540] Li Y. Sun Y. Chen M. (2021). An evaluation of the impact of monetary easing policies in times of a Pandemic. Public Health Frontier, 8, 627001. 10.3389/fpubh.2020.627001 PMC7841454 33520925

[bibr61-21582440231181540] Mäkelä P. Huhtanen P. (2010). The effect of survey sampling frame on coverage: The level of and changes in alcohol-related mortality in Finland as a test case. Addiction, 105(11), 1935–1941. 10.1111/j.1360-0443.2010.03069.x 21040059 PMC3058593

[bibr62-21582440231181540] Makun K. Jayaraman T. K. (2021). COVID-19 impact on remittances and economic growth in three transitional countries in ASEAN: Evidence from nonlinear analysis. Economic Bulletin, 41(3), 1566–1578.

[bibr63-21582440231181540] McCabe S. E. Kloska D. D. Veliz P. Jager J. Schulenberg J. E. (2016). Developmental course of nonmedical use of prescription drugs from adolescence to adulthood in the united states: National longitudinal data. Addiction, 111(12), 2166–2176. 10.1111/add.13504 27338559 PMC5183528

[bibr64-21582440231181540] McKibbin W. Fernando R. (2020). The Global Macroeconomic Impacts of COVID-19: Seven Scenarios (CAMA Working Paper No. 19/2020). https://ssrn.com/abstract=3547729 or 10.2139/ssrn.3547729

[bibr65-21582440231181540] McNeish D. Stapleton L. M. (2016). Modeling clustered data with very few clusters. Multivariate behavioral research, 51(4), 495–518. 10.1080/00273171.2016.1167008 27269278

[bibr66-21582440231181540] McNeish D. M. (2016). Using data-dependent priors to mitigate small sample bias in latent growth models a discussion and illustration using Mplus. Journal of Educational and Behavioral Statistics, 41(1), 27–56. 10.3102/1076998615621299

[bibr67-21582440231181540] Miočević M. MacKinnon D. P. Levy R. (2017). Power in Bayesian mediation analysis for small sample research. Structural Equation Modeling, 24(5), 666–683. 10.1080/10705511.2017.1312407 29662296 PMC5898829

[bibr68-21582440231181540] Morgan P. J. Trinh L. Q. (2021). Impacts of COVID-19 on households in ASEAN countries and their implications for Human Capital Development (ADBI working paper no 1226), Asian Development Bank Institute. https://www.adb.org/publications/impacts-covid-19-households-asean-countries

[bibr69-21582440231181540] Muthén B. Asparouhov T. (2012). Bayesian structural equation modeling: A more flexible representation of substantive theory. Psychological Methods, 17(3), 313–335. 10.1037/a0026802 22962886

[bibr70-21582440231181540] Muthén B. O. Curran P. J. (1997). General longitudinal modeling of individual differences in experimental designs: A latent variable framework for analysis and power estimation. Psychological Methods, 2, 371–402. 10.1037/1082-989x.2.4.371

[bibr71-21582440231181540] Najabat A. Xuhua H. Jamal H. Memoona N. (2021). Assessing the environmental impacts of COVID-19; A review. Polish Journal of Environmental Studies, 30(5), 4401–4403. 10.15244/pjoes/130337

[bibr72-21582440231181540] Narayan P. K. (2020). Oil price news and COVID-19—Is there any connection? Energy Research Letters, 1(1), 13176. 10.46557/001c.13176

[bibr73-21582440231181540] Natesan P. (2015). Comparing interval estimates for small sample ordinal CFA models. Frontiers in Psychology, 6, 1599. 10.3389/fpsyg.2015.01599 26579002 PMC4626630

[bibr74-21582440231181540] Padhan R. Prabheesh K. P. (2021). The economics of COVID-19 pandemic: A survey. Economic Analysis and Policy, 70, 220–237. 33658744 10.1016/j.eap.2021.02.012 PMC7906538

[bibr75-21582440231181540] Peeters M. Monshouwer K. Janssen T. Wiers R. W. Vollebergh W. A. (2014). Working memory and alcohol use in at-risk adolescents: A 2-year follow-up. Alcoholism Clinical and Experimental Research, 38(4), 1176–1183. 10.1111/acer.12339 24460848

[bibr76-21582440231181540] Phan D. H. B. Narayan P. K. (2020). Country responses and the reaction of the stock market to COVID-19—A preliminary exposition. Emerging Markets Finance and Trade, 56(10), 2138–2150. 10.1080/1540496X.2020.1784719

[bibr77-21582440231181540] Polemis M. Soursou S. (2020). Assessing the impact of the COVID-19 pandemic on the greek energy firms: An event study analysis. Energy Research Letters, 1(3), 17238. 10.46557/001c.17238

[bibr78-21582440231181540] Price L. R. (2012). Small sample properties of Bayesian multivariate autoregressive time series models. Structural Equation Modeling, 19(1), 51–64.

[bibr79-21582440231181540] Purnomo E. P. Nurmandi A. Dewi A. Rosa E. M. Bayu A. H. Erviana R. (2022). ASEAN policy responses to COVID-19 pandemic: Adaptation and experimentation policy: A study of ASEAN countries policy volatility for COVID-19 pandemic. Sage Open, 12(1), 1–10. 10.1177/21582440221082145

[bibr80-21582440231181540] Qin X. Huang G. Shen H. Fu M. (2020). COVID-19 pandemic and firm-level cash holding—Moderating effect of goodwill and goodwill impairment. Emerging Markets Finance and Trade, 56(10), 2243–2258. 10.1080/1540496X.2020.1785864

[bibr81-21582440231181540] Rietbergen C. Debray T. P. A. Klugkist I. Janssen K. J. M. Moons K. G. M. (2017). Reporting of Bayesian analysis in epidemiologic research should become more transparent. Journal of Clinical Epidemiology, 86, 51–58.e2. 10.1016/j.jclinepi.2017.04.008 28428139

[bibr82-21582440231181540] Rizvi S. A. R. Juhro S. M. Narayan P. K. (2021). Understanding market reaction to Covid-19 monetary and fiscal stimulus in major asean countries. Buletin Ekonomi Moneter dan Perbankan, 24(3), 313–334. 10.21098/bemp.v24i3.1690

[bibr83-21582440231181540] Roberts G. O. Rosenthal J. S. (2001). Optimal scaling for various Metropolis-Hastings algorithms. Statistical Science, 16, 351–367.

[bibr84-21582440231181540] Roe-Sepowitz D. (2009). Comparing male and female juveniles charged with homicide: Child maltreatment, substance abuse, and crime details. Journal of Interpersonal Violence, 24(4), 601–617. 10.1177/08862605083172 18487523

[bibr85-21582440231181540] Serang S. Zhang Z. Helm J. Steele J. S. Grimm K. J. (2015). Evaluation of a Bayesian approach to estimating nonlinear mixed-effects mixture models. Structural Equation Modeling, 22(2), 202–215. 10.1080/10705511.2014.937322

[bibr86-21582440231181540] Sharma S. S. (2020). A note on the Asian market volatility during the COVID-19 pandemic. Asian Economics Letters, 1(2), 17661. 10.46557/001c.17661

[bibr87-21582440231181540] Smid S. C. McNeish D. Miočević M. van de Schoot R. (2020). Bayesian versus frequentist estimation for structural equation models in small sample contexts: A systematic review. Structural Equation Modeling, 27(1), 131–161. 10.1080/10705511.2019.1577140

[bibr88-21582440231181540] Thach N. N. Ha D. T. Trung N. D. Kreinovich V. (2022). Prediction and causality in econometrics and related topics (Vol. 983). Springer.

[bibr89-21582440231181540] Thach N. N. Kreinovich V. Ha D. T. Trung N. D. (2022). Financial econometrics: Bayesian analysis, quantum uncertainty, and related topics (Vol. 427). Springer.

[bibr90-21582440231181540] Thach N. N. Kreinovich V. Trung N. D. eds (2021). Data Science for financial econometrics (Vol. 898). Springer.

[bibr91-21582440231181540] Thach N. N. Linh N. T. X. Hac L. D. Ngoc L. T. B. Hai D. H. (2022). Specific macro factors affecting economic growth during the COVID-19 pandemic : Evidence from EAGLEs. Indian Journal of Finance, 16(3), 8–27. 10.17010/ijf/2022/v16i3/168700

[bibr92-21582440231181540] Topcu M. Gulal O. S. (2020). The impact of COVID-19 on emerging stock markets. Finance Research Letters, 36, 101691. 10.1016/j.frl.2020.101691 32837378 PMC7348595

[bibr93-21582440231181540] van der Lee J. H. Wesseling J. Tanck M. W. Offringa M. (2008). Efficient ways exist to obtain the optimal sample size in clinical trials in rare diseases. Journal of Clinical Epidemiology, 61(4), 324–330. 10.1016/j.jclinepi.2007.07.008 18313556

[bibr94-21582440231181540] van de Schoot R. Broere J. J. Perryck K. H. Zondervan-Zwijnenburg M. van Loey N. E. (2015). Analyzing small data sets using Bayesian estimation: The case of posttraumatic stress symptoms following mechanical ventilation in burn survivors. European Journal of Psychotraumatology, 6, 25216. 10.3402/ejpt.v6.25216 Article 25216. 25765534 PMC4357639

[bibr95-21582440231181540] van de Schoot R. Winter S. D. Ryan O. Zondervan-Zwijnenburg M. Depaoli S. (2017). A systematic review of Bayesian articles in psychology: The last 25 years. Psychological Methods, 22(2), 217–239. 10.1037/met0000100 28594224

[bibr96-21582440231181540] van Erp S. Mulder J. Oberski D. L. (2018). Prior sensitivity analysis in default bayesian structural equation modeling. Psychological Methods, 23(2), 363–388. 10.1037/met0000162 29172613

[bibr97-21582440231181540] Wagenmakers E. J. Lee M. Lodewyckx T. Iverson G. J. (2008). Bayesian versus frequentist inference. In Hoijtink H. Klugkist I. Boelen P. (Eds.), Bayesian evaluation of informative hypotheses (pp. 181–207). Springer Science + Business Media.

[bibr98-21582440231181540] World Bank. (2022). World Development Indicators. http://datatopics.worldbank.org/worlddevelopment-indicators/.

[bibr99-21582440231181540] Worldometer. (2021). Coronavirust Cases. https://www.worldometers.info/coronavirus/

[bibr100-21582440231181540] Yuan Y. MacKinnon D. P. (2009). Bayesian mediation analysis. Psychological Methods, 14(4), 301–322. 10.1037/a0016972 19968395 PMC2885293

[bibr101-21582440231181540] Zondervan-Zwijnenburg M. Depaoli S. Peeters M. van de Schoot R. (2018). Pushing the limits: The performance of maximum likelihood and Bayesian estimation with small and unbalanced samples in a latent growth model. Methodology, 1(1), 1–13. 10.1027/1614-2241/a000162

[bibr102-21582440231181540] Zondervan-Zwijnenburg M. Peeters M. Depaoli S. Van de Schoot R. (2017). Where do priors come from?. osf.io/aw8fy.

